# Properties analysis of transcription factor gene *TasMYB36* from *Trichoderma asperellum* CBS433.97 and its heterogeneous transfomation to improve antifungal ability of *Populus*

**DOI:** 10.1038/s41598-017-13120-w

**Published:** 2017-10-09

**Authors:** Shida Ji, Zhiying Wang, Jinjie Wang, Haijuan Fan, Yucheng Wang, Zhihua Liu

**Affiliations:** 1 School of Forestry, Northeast Forestry University, 26 Hexing Road, 150040 Harbin, China; 2 Key Laboratory of Biogeography and Bioresource in Arid Land, Xinjiang Institute of Ecology and Geography, Chinese Academy of Sciences, Urumqi, 830011 China

## Abstract

The transcription of *TasMYB36* in the biocontrol species *T. asperellum* was upregulated in four different pathogenic fermentation broths, suggesting that *TasMYB36* plays an important role in the response to biotic stresses. Seventy-nine MYB transcription factors that were homologous to TasMYB36 from six sequenced *Trichoderma* genomes were analyzed. They were distributed in fourteen clades in the phylogenetic tree. The 79 MYBs contained 113 DNA binding domains, and their amino acid sequences were conserved and were different to those in plants. The promoters of 79 *MYBs* contained 1374 cis-regulators related to the stress response, such as GCR1 (17.5%) and GCN4 (15.5%). Subsequently, *TasMYB36* was integrated into the genome of *Populus davidiana* × *P. alba* var. *pyramidalis* (PdPap poplar), and after co-culture of the transformants (PdPap-TasMYB36s) with *Alternaria alternate*, the transcription of genes in the jasmonic acid (JA) and salicylic acid (SA) hormone signaling pathways were upregulated; the POD, SOD and CAT activities were enhanced; and the reactive oxygen content was reduced in PdPap-TasMYB36s. The disease spots area on PdPap-TasMYB36s leaves infected by *A. alternate* were average 0.63% (PdPap-Con: 24.7%). In summary, *TasMYB36* of *T. asperellum* CBS433.97 is an important defense response gene that upregulates other stress response genes and could improve resistance to biotic stresses.

## Introduction

*Trichoderma* spp. are distributed widely in soil and can colonize plant roots to form rhizospheric microbiota^1–3^. They are considered as biocontrol agents for foliar^4^ and soil-borne diseases^5,6^, and have been used to control certain phytopathogens *in vitro* and in the field. The mechanisms of their anti-phytopathogen activities include mycoparasitism^7^, inducing resistance^8,9^, niche exclusion competition, and plant growth promotion^10,11^, which is achieved by upregulating the expression of resistance genes and synthesizing bioactive compounds, such as enzymes and antibiotics^12^. MYB transcription factors are believed to play an important role in the anti-phytopathogen functions and survival of *Trichoderma* spp.

MYB transcription factors are essential regulators of gene transcription, and exist widely in animals, plants, and fungi^13^. In plants, MYB transcription factors are characterized by a highly conserved MYB DNA-binding domain, and are classified into four major groups, 1R-MYB, 2R-MYB, 3R-MYB, and 4R-MYB, based on the number and position of MYB repeats^14^. The three-dimensional structure of the MYB domain showed that the DNA recognition site α-helix interacts with the major groove of DNA^15^. MYB proteins can interact with specific DNA sequences, for example, many R2R3-MYB transcription factors bind to DNA motifs that are enriched in adenosine (A) and cytosine (C) residues^16,17^, where guanine (G) residues are either absent or depleted^18,19^.

MYB transcription factors are involved in development, secondary metabolism, hormone signal transduction, abiotic stress tolerance, and disease resistance (Katiyar *et al*.^20^). Many *MYB* genes in plants had been studied. For hormone signal transduction, the effects of AtMYB44 in salicylic acid (SA) and jasmonic acid (JA)-mediated defense responses are achieved through direct regulation of WRKY70 expression^21^. MYB72 is required in the early signaling steps of rhizobacteria-induced systemic resistance in *Arabidopsis* ^22^. The upregulation of *MYBs* under *Puccinia striiformis* stress indicated that *MYBs* might play an important role in conferring resistance to stripe rust in wheat^23^. A sorghum *MYB* transcription factor enhanced resistance to leaf blight in Maize through the biosynthesis of 3-deoxyanthocyanidin phytoalexins at the site of primary infection^24^. R2R3 MYB transcription factor from wheat, TaPIMP1, mediated host resistance to *Bipolaris sorokiniana* ^24^. Expression of a wheat MYB gene in transgenic tobacco enhanced resistance to *Ralstonia solanacearum*, and to drought and salt stresses^25^. Transgenic wheat expressing *Thinopyrum intermedium* MYB transcription factor TiMYB2R-1 showed enhanced resistance to take-all disease caused by the fungus *Gaeumannomyces graminis var. tritici* ^26^. However, few *MYB* genes in fungi have been studied. In the only reported study, in a *Saccharomyces cerevisiae* insertional mutant, the *BAS1* gene, a member of the MYB family of transcription factors, was inactivated, which resulted in a delay in the germination of the spores and an abnormally prolonged trophic phase^27^.

To the best of our knowledge, there have been no reports related to MYB transcription factors in biocontrol *Trichoderma* species. *Trichoderma* spp. and plants survive in the same environments and stresses, therefore, their resistance genes might show a tendency for convergent evolution. And previous and our study showed the primary structure of MYB transcription factors in plant and *Trichoderma* is quite similar^28,29^, so they might share the similar functions, just like the MYB in plants, those in *Trichoderma* might also enhance resistance to biotic stresses. In the present study, the transcription levels of *TasMYB36* in *T. asperellum* CBS433.97 under five phytopathogen stresses were detected using quantitative real-time reverse transcription PCR (qRT-PCR). To identify the homologous genes of *TasMYB36*, 79 *MYB* genes were obtained from six sequenced *Trichoderma* genomes, including *T. asperellum* CBS433.97, *T. harzianum* CBS226.95, *T. virens* Gv29-8, *T. atroviride*, *T. reesei*, and *T. longibrachiatum* ATCC18648. The properties of their coding regions and promoters were analyzed. Multiple sequence alignment and phylogenetics analyses were conducted. In addition, following its heterologous transformation into *Populus davidiana* × *P. alba* var. *pyramidalis* (PdPap poplar), the transcription of *TasMYB36* in the transformants (PdPap-TasMYB36s) was detected using qRT-PCR. After co-culture of PdPap-TasMYB36s with the phytopathogen *Alternaria alternate*, the transcription of genes related to JA and SA hormone signal transduction pathways were analyzed, and the reductase activities (POD, SOD, and CAT) and the reactive oxygen species (ROS) contents in the PdPap-TasMYB36s were detected. Finally, the antifungal capabilities of PdPap-TasMYB36s were detected by infecting the leaves of the poplar transformants with the 5 × 10^6^ spores/mL of *A. alternate*.

## Results

### Cloning of the *TasMYB36* gene from *T. asperellum* CBS433.97 and bioinformatics analysis

The *TasMYB36* gene was cloned from the *T. asperellum* CBS433.97 genome. Its DNA is 1146 bp in length and contains one intron. Its cDNA is 996 bp in length and encodes a protein comprising 331 aa. The isoelectric point (pI) of TasMYB36 protein is 10.25 and its molecular weight is 36.0 kDa. The protein was identified as an MYB transcription factor (Pfam 00249) using Pfam prediction, with one MYB DNA-binding domain at amino acids 15 to 57. The cDNA sequence was deposited in GenBank with the accession number KT834975.

### Differential expression of *TasMYB36* in response to five phytopathogen fermentation broths

**Figure 1:**
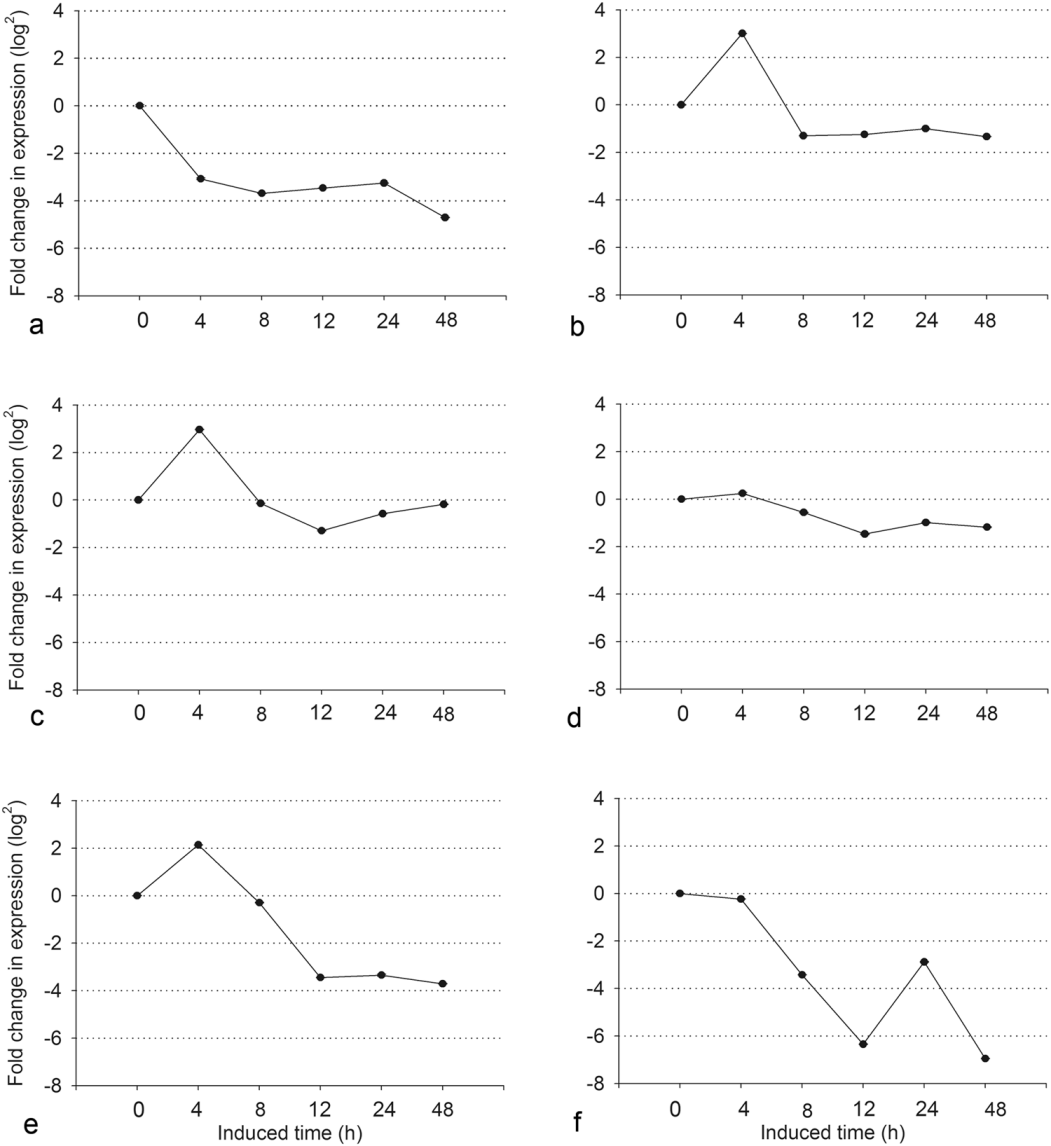
The stress response of *TasMYB36* of *Trichoderma* against five pathogen fermentation broths. (**a**) Mineral medium (MM) with 0.1% (w/v) glucose (as the control); (**b**–**f**) Five biotic stresses (5% (v/v) fermentation broths from *A. alternate*, *C. chrysosperma*, *F. oxysporum*, *R. solani*, and *S. sclerotiorum*).

The response of *TasMYB36* was investigated by qRT-PCR after *T. asperellum* CBS433.97 was subjected to five biotic stresses; MM without phytopathogens was used as a control (Fig. 1a). *TasMYB36* transcription was upregulated strongly at 4 h in response to the fermentation broths of *A. alternate* (Fig. 1b) and *Cytospora chrysosperma* (Fig. 1c), by 8.06 (2^3.01^) and 7.78 (2^2.96^)-fold, respectively. *TasMYB36* transcription was also upregulated at 4 h in response to the fermentation broths of *Fusarium oxysporum* (Fig. 1d) and *Rhizoctonia solani* (Fig. 1e), by 1.18 (2^0.24^) and 4.41 (2^2.14^)-fold, respectively. However, *TasMYB36* transcription was downregulated by the fermentation broths of *Sclerotinia sclerotiorum* (Fig. 1f), but was downregulated more strongly byMM at 4h, reaching to 8.46 (2^3.08^)-fold. These results suggested that the *TasMYB36* gene is closely associated with the response of *T. asperellum* CBS433.97 against biotic stresses.

### The properties of 79 MYB transcription factors

The properties of 79 MYB transcription factors from six sequenced *Trichoderma* genomes were analyzed (Table 1). There were 14, 13, 12, 12, 15, and 13 MYB transcription factors being encoded by *T. asperellum* CBS433.97, *T. harzianum* CBS 226.95, *T. virens* Gv29-8, *T. atroviride*, *T. reesei*, and *T. longibrachiatum* ATCC18648, respectively. Fifteen *MYB* genes had no intron and the other *MYB* genes contained different numbers of introns (1–7). The pIs of MYB transcription factors showed large differences, ranging from 4.04 to 10.6, and there were totally 31 basic proteins (pI > 7) and 48 were acidic proteins (pI < 7). Their molecule weights ranged from 22.21 to 255.29 kDa. The 79 MYB transcription factors were classified into four major groups, including 1R-MYB, 2R-MYB, 3R-MYB, and 4R-MYB, based on the repeat numbers of their MYB DNA binding domains^14^. All 79 MYB transcription factors from six sequenced *Trichoderma* genomes contained 48 1R-MYB, 30 2R-MYB and only 1 3R-MYB (in *T. harzianum*), without 4R-MYB (Table 1). According the type and location of the MYB DNA binding domains, the MYB transcription factors could be divided in nine types, including Type B (n = 31), Type B_2_ (n = 2), Type B_6_ (n = 8), Type B_7_ (n = 6), Type BB (n = 10), Type B_6_B (n = 12), Type BB_6_ (n = 6), Type B_6_B_4_ (n = 3), and Type B_6_B_6_B (n = 1). Most 1R-MYB transcription factors belonged to Type B and most 2R-MYB transcription factors belonged to Type BB and Type B_6_B (Table 1).

**Table 1:** The properties of 79 MYB transcription factors from 6 sequenced *Trichoderma* genomes.

Gene Name	The Location of Gene	Introns	AA	pI	MW (kDa)	CD	Type
TasMYB25	scaffold_11:371466–372557 (−)	3	231	9.65	25.26	135–177/179–222	
TasMYB27	scaffold_11:1343526–1344021 (−)	1	238	5.78	27.40	129–186/176–207	
TasMYB36	scaffold_4:2116156–2117302 (−)	1	331	10.2	36.62	15–57	
TasMYB38	scaffold_20:103014–103337 (−)	0	349	10.3	38.11	42–88/98–134	
TasMYB58	scaffold_6:1378471–1380145 (+)	1	523	6.64	58.88	79–126	
TasMYB62	scaffold_4:1210975–1213105 (−)	0	570	6.09	62.64	414–501	
TasMYB67	scaffold_2:1420902–1422857 (−)	1	621	9.07	67.27	306–356/454–503	
TasMYB75	scaffold_8:12464–15001 (−)	2	690	4.92	75.36	393–436	
TasMYB86	scaffold_2:2509057–2511447 (−)	1	777	6.32	86.69	8–52/54–106	
TasMYB92	scaffold_4:377301–380443 (−)	2	816	5.12	92.69	113–173	
TasMYB118	scaffold_8:586973–590579 (−)	4	1089	5.33	118.05	591–647	
TasMYB171	scaffold_3:1189064–1195065 (−)	6	1515	5.54	171.69	1079–1139/1147–1238	
TasMYB182	scaffold_1:2715111–2722223 (+)	5	1657	8.99	182.38	671–715	
TasMYB255	scaffold_4:2100911–2107305 (−)	1	2111	6.46	255.29	989–1032/1280–1323	
ThaMYB22	scaffold_7:2283613–2284276 (−)	1	195	5.22	22.11	86–143/134–164	
ThaMYB28	scaffold_18:68586–70065 (−)	5	263	9.21	28.85	162–206/208–253	
ThaMYB34	scaffold_12:1162369–1163533 (−)	4	301	7.65	34.72	13–76/68–127/116–162	
ThaMYB35	scaffold_4:2044751–2045862 (−)	1	326	10.4	35.88	13–57	
ThaMYB36	scaffold_9:184232–184780 (+)	0	330	7.24	36.56	10–56/62–107	
ThaMYB58	scaffold_10:864556–866677 (−)	1	523	6.47	58.95	79–126	
ThaMYB62	scaffold_4:1415189–1416886 (+)	0	565	5.88	62.38	407–494	
ThaMYB75	scaffold_30:20305–22916 (−)	2	691	4.90	75.22	392–437	
ThaMYB68	scaffold_1:2082037–2083923 (−)	0	628	8.75	68.31	320–370/475–539	
ThaMYB86	scaffold_1:3125036–3127439 (+)	1	777	5.72	86.83	9–69/58–102	
ThaMYB113	scaffold_14:406732–410162 (−)	4	1049	5.37	113.88	582–638	
ThaMYB173	scaffold_5:2004340–2010727 (+)	5	1542	5.32	173.62	1118–1178	
ThaMYB239	scaffold_4:2029596–2037045 (−)	2	2145	6.91	239.70	1289–1334	
TviMYB26	scaffold_20:475405–476732(−)	2	244	9.37	26.69	164–206	
TviMYB27	scaffold_11:861800–862578 (−)	1	237	5.75	27.03	128–185/174–220	
TviMYB36	scaffold_2:600988–602083 (+)	1	328	10.4	36.34	13–57	
TviMYB37	scaffold_21:527963–529148 (−)	1	340	8.73	37.46	91–141	
TviMYB42	scaffold_4:2202176–2203303 (−)	0	375	8.80	42.03	43–89/95–135	
TviMYB57	scaffold_2:1224262–1225998 (−)	1	515	7.77	57.27	356–443	
TviMYB61	scaffold_1:2235161–2236948 (−)	1	563	5.56	61.49	286–336/432–496	
TviMYB70	scaffold_1:2459037–2460905 (−)	0	622	4.35	70.28	130–175	
TviMYB73	scaffold_23:451456–453932 (+)	2	666	4.97	73.01	367–412	
TviMYB86	scaffold_1:3347583–3349985 (+)	1	777	5.71	86.60	9–69/60–102	
TviMYB110	scaffold_6:98720–101772 (−)	1	993	4.93	110.71	573–633	
TviMYB243	scaffold_2:609834–616508 (+)	2	2175	7.76	243.66	1304–1349	
TreMYB27	scaffold_9:1030437–1031225 (−)	1	235	5.87	27.03	126–183/172–218	
TreMYB32	scaffold_1:1815585–1816698 (+)	2	292	10.3	32.37	13–57	
TreMYB34	scaffold_5:317936–320047 (+)	4	318	8.94	34.45	164–208	
TreMYB38	scaffold_10:782895–784125 (+)	2	349	8.99	38.32	96–166	
TreMYB40	scaffold_28:181570–182670 (−)	0	336	9.62	40.30	43–89/95–135	
TreMYB56	scaffold_1:767149–768965 (+)	1	514	9.08	56.63	355–442	
TreMYB58	scaffold_24:241032–243046 (−)	1	514	6.29	58.95	79–126	
TreMYB70	scaffold_2:333659–335521 (−)	0	620	4.43	70.36	133–178	
TreMYB72	scaffold_2:61770–64535 (−)	0	669	6.57	72.02	363–413/510–574	
TreMYB73	scaffold_2:1994423–1996847 (+)	3	661	5.07	72.19	361–406	
TreMYB86	scaffold_2:1189091–1191556 (+)	1	777	6.01	86.34	9–69/58–102	
TreMYB109	scaffold_26:242436–245500 (+)	1	988	5.07	109.39	566–626/614–718	
TreMYB125	scaffold_11:581897–585735 (−)	5	1144	6.23	125.58	133–178	
TreMYB186	scaffold_5:1293771–1300234 (−)	7	1074	9.03	186.65	738–783	
TreMYB232	scaffold_1:1826274–1833439 (+)	5	2095	6.19	232.59	1018–1063	
TatMYB27	scaffold_7:1531759–1533595 (+)	7	249	6.45	27.53	140–184	
TatMYB28	scaffold_17:70061–70858 (+)	1	240	5.85	27.68	131–188/177–223	
TatMYB33	scaffold_16:754009–754332 (−)	0	305	10.6	33.74	9–55/61–106	
TatMYB36	scaffold_12:678408–679586 (−)	2	326	10.3	36.24	13–57	
TatMYB37	scaffold_6:756022–757214 (−)	1	336	9.04	37.82	88–156	
TatMYB56	scaffold_10:1254042–1256117 (−)	1	517	9.02	56.12	351–438	
TatMYB65	scaffold_2:1998371–2000164 (+)	0	597	9.33	65.11	331–381/483–547	
TatMYB72	scaffold_7:9103–11290 (−)	2	663	4.97	72.75	364–409	
TatMYB86	scaffold_2:841485–843878 (−)	1	777	6.36	86.50	9–69/58–102	
TatMYB163	scaffold_5:1479192–1484396 (−)	5	1497	6.78	163.54	913–984	
TatMYB167	scaffold_3:1802095–1808280 (+)	7	1487	5.49	167.95	1084–1144	
TatMYB241	scaffold_6:756022–757214 (−)	1	2176	5.81	241.48	1063–1108/1354–1399	
TloMYB24	scaffold_8:1490083–1490650 (−)	1	263	5.66	24.11	103–160/149–196	
TloMYB29	scaffold_1:2981637–2982199 (−)	4	265	9.06	29.15	165–209/211–256	
TloMYB41	scaffold_3:2453950–2455526 (−)	0	375	9.76	41.20	43–89/95–135	
TloMYB58	scaffold_6:1035137–1037465 (−)	1	520	6.29	58.49	79–126	
TloMYB63	scaffold_3:1526425–1528647 (−)	0	582	6.16	63.42	423–510	
TloMYB67	scaffold_2:2077891–2080313 (+)	2	609	6.04	67.27	9–69/58–102	
TloMYB75	scaffold_2:2857988–2860562 (+)	2	690	4.04	75.04	391–436	
TloMYB82	scaffold_2:1041805–1044054 (−)	0	749	6.56	82.31	368–418/520–584	
TloMYB95	scaffold_6:296503–299477 (−)	2	848	5.34	95.92	111–171	
TloMYB104	scaffold_14:670920–674674 (−)	3	1013	5.26	138.83	472–528	
TloMYB109	scaffold_5:1468188–1471776 (−)	1	998	5.09	109.62	565–625/614–718	
TloMYB138	scaffold_1:2050016–2056293 (+)	3	1259	8.97	138.85	288–333	
TloMYB244	scaffold_3:536391–543984 (+)	2	2183	8.94	244.62	1310–1355	

: MYB DNA-bind, : MYB DNA-bind 2,  MYB DNA-bind 4, : MYB DNA-bind 6, : MYB DNA-bind 7. : Type1(B), : Type2(B2), : Type3(B_6_), : Type4(B7) : Type5(BB), : Type6(B_6_B), : Type7(BB_6_),  Type8(B_6_B_4_) : Type9(B_6_B_6_B), CD: conserved domain. AA: amino acid. MW: molecular weight.

### The phylogenetics and alignment analysis of 79 MYB transcription factors

**Figure 2:**
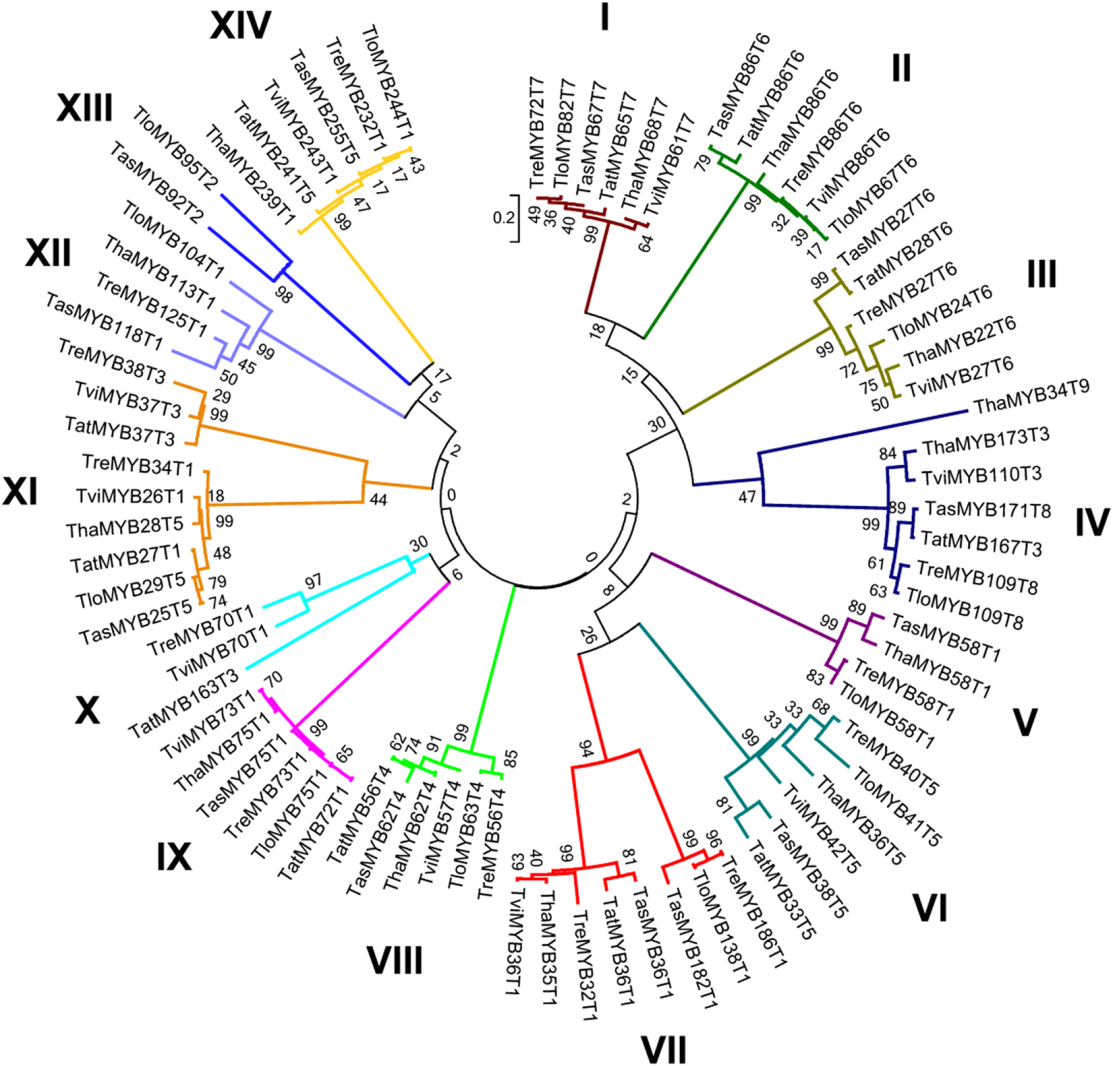
Phylogenetic analysis of 79 MYB transcription factors. The 79 MYB transcription factors were obtained from six sequenced *Trichoderma* genomes, including *T. asperellum* CBS433.97, *T. harzianum* CBS 226.95, *T. virens* Gv29-8, *T. atroviride*, *T. reesei*, and *T. longibrachiatum* ATCC18648. They contained 14, 13, 12, 12, 15, and 13 MYB transcription factors, respectively. The phylogenetic tree was constructed using the neighbor-joining method in the MEGA6.0 program.

The phylogenetic analysis divided the 79 MYB transcription factors into 14 clades and the TasMYB36 protein studied in this paper was placed in Clade VII (Fig. 2). And the genetic distances of the 79 MYB transcription factors that occurs within each *Trichoderma* specie was calculated (Supplemental Table 2). For example, in the *T. asperellum*, TasMYB36 and TasMYB182 have the closest genetic relationship, the value was 1.223 (Supplemental Table 2), and TasMYB118 and TasMYB171 have the most remote genetic relationship, the value was 2.885 (Supplemental Table 2), the genetic relationship of different MYB transcription factors was consistent with their distribution in the phylogenetic tree (Fig. 2). The distribution of MYB proteins in the phylogenetic tree was closely related to their type and molecular weight. Most clades contained one MYB Type. Clades X and XIV contained two MYB Types, and clades XI and IV contained three MYB Types (Fig. 2). The MYB transcription factors only containing “MYB DNA-bind 2” or “MYB DNA-bind 7” were distributed in Clades XIII and VIII, respectively. The MYB transcription factors containing “MYB DNA-bind 4” were distributed in Clade IV. MYB proteins in the same clade shared the similar molecular weight, for example, six MYB proteins in the Clades XIV had the highest molecular weight (average 242.3kDa), and six MYB proteins in the Clades III had the lowest molecular weight (average 25.8kDa). MYB transcription factors in each clade were derived from different *Trichoderma* spp., but shared similar pI and number of introns (Table 1), Most MYB transcription factors in clade VI had no introns, but those in clades II, III, V, and XIII had one or two introns (Table 1). MYB transcription factors in clades II, III, IV, V, IX, X, XII, and XIII (Fig. 2) were acidic proteins (Table 1) and the pI of proteins in clade IX was the lowest (average 4.81 ± 0.77). MYB transcription factors in clades VI, VII, and XI (Fig. 2) were basic proteins (Table 1) and the pIs of proteins in clade VII were the highest, (average 9.82 ± 0.85).

**Figure 3:**
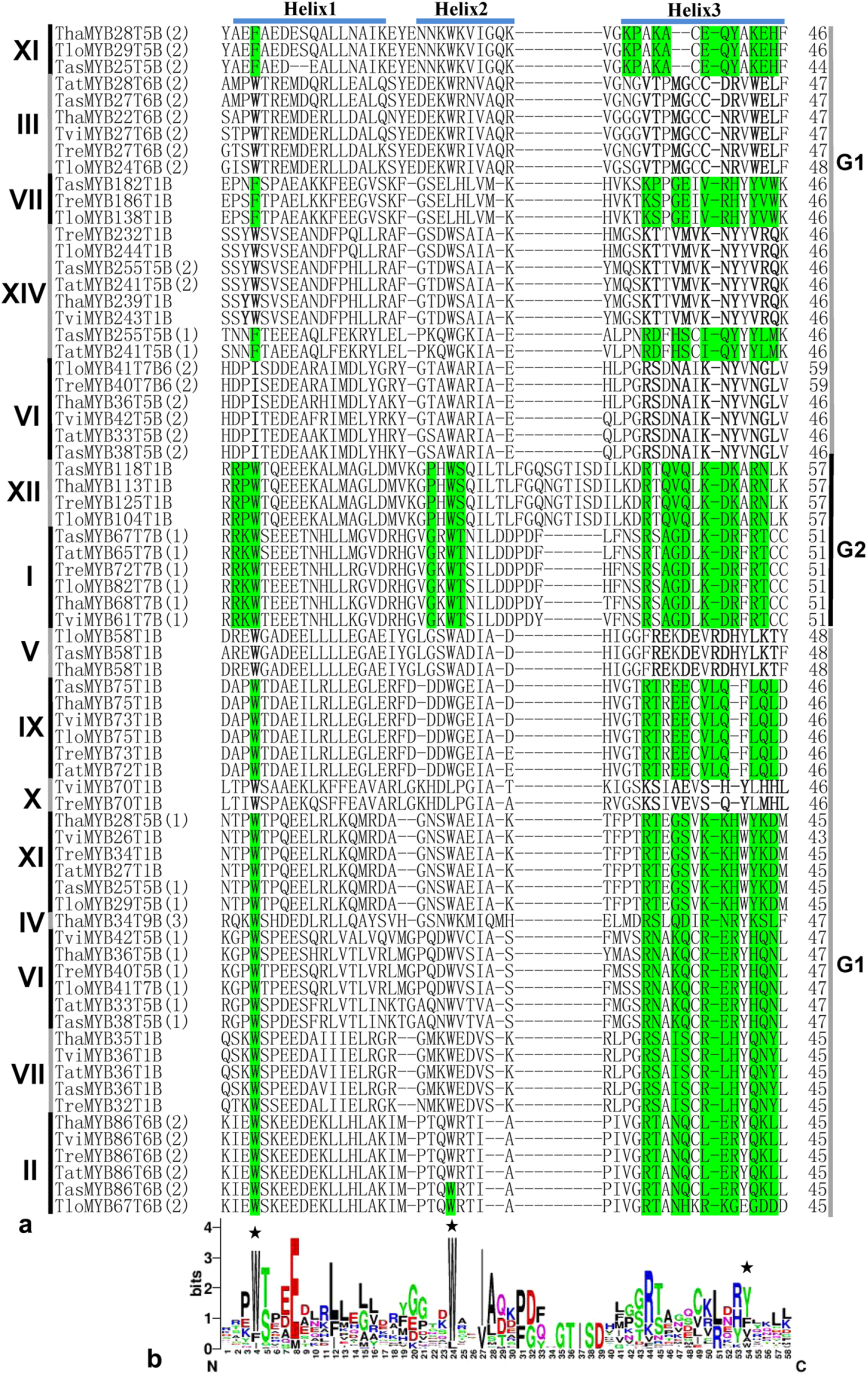
Alignment analysis of 72 “MYB DNA-bind”. The roman numerals indicate the location of the corresponding MYB proteins in the phylogenetic tree (Fig. 2). The DNA binding sites were predicted using BlastP at NCBI and are highlighted in green; however, the DNA binding sites of other “MYB DNA-bind” were not obtained; therefore, these DNA binding sites were predicted according to previous results and are highlighted in bold. “MYB DNA-bind” was divided in two groups and marked as G1 and G2.

**Figure 4:**
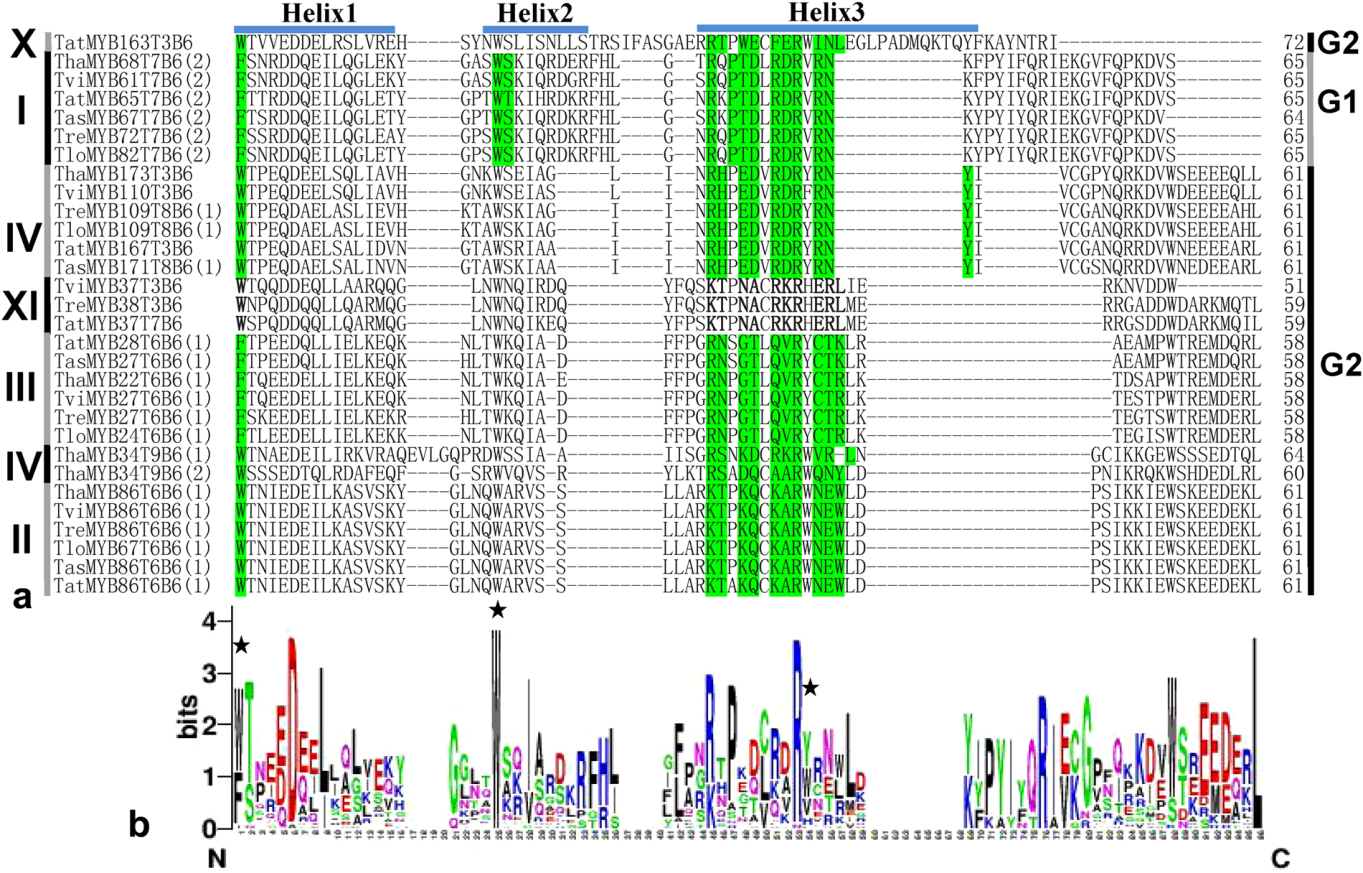
Alignment analysis of 31 “MYB DNA-bind 6”. The roman numerals indicate the location of the corresponding MYB proteins in the phylogenetic tree (Fig. 2). The DNA binding sites were predicted using BlastP at NCBI and are highlighted in green; however, the DNA binding sites of other “MYB DNA-bind 6” were not obtained; therefore, these DNA binding sites were predicted according to previous results and are highlighted in Bold. “MYB DNA-bind 6” was divided in two groups and was marked as G1 and G2.

The 79 MYB transcription factors contained 113 MYB DNA binding domains, which were classified into five categories, including “MYB DNA-bind” (n = 72), “MYB DNA-bind 2” (n = 2), “MYB DNA-bind 4” (n = 3), “MYB DNA-bind 6” (n = 30), and “MYB DNA-bind 7” (n = 6). The proteins contained many more “MYB DNA-bind” and “MYB DNA-bind 6” domains than the other domains, and the alignment analysis showed the DNA-binding sites (highlighted in green or Bold) of MYB transcription factors in the same clade (Figs 3 and 4) were relatively conserved; however, those in different clades had obvious differences. According the distribution of DNA binding sites, the “MYB DNA-bind” domains were classified into two groups. The DNA binding sites of “MYB DNA-bind” in Group 1 contained 15 residues and there were three, three, and nine residues in α-helix1, α-helix2, and α-helix3, respectively. Blastp analysis at NCBI showed that the fourth amino acid of the DNA binding site was located in α-helix2 and was a basic amino acid; the seventh, tenth, twelfth, and thirteenth amino acids of the DNA binding site were located in α-helix3 and were also basic amino acid; and the eleventh amino acid was aspartic acid (D). The DNA binding sites of “MYB DNA-bind” in Group 2 contained 11 residues and there were 1 and 10 residues in α-helix1 and α-helix3, respectively (Fig. 3). The MYB transcription factors containing “MYB DNA-bind” in Group 1 were distributed in Clades I and XII of the phylogenetic tree (Fig. 2), which contained binding sites “RKW/G-WT/R-AGD-KDR-RT” and “RPW/P-WS/R-QVQ-KDK-RN”, respectively. The MYB transcription factors containing “MYB DNA-bind” in Group 2 were distributed in Clades II, III, IV, V, VI, VII, X, XI, and XIV (Fig. 2).

The “MYB DNA-bind 6” domains were also classified into two groups (Fig. 4). The DNA binding sites of “MYB DNA-bind 6” in Group 1 contained 12 residues and there were one, two, and nine residues in α-helix1, α-helix2, and α-helix3, respectively. Blastp analysis at NCBI showed that the second amino acid in front of conserved “W” residue in α-helix2 was a basic amino acid; the fourth, eighth, tenth, and eleventh amino acids of DNA binding sites were arginine (pI < 7) located in α-helix3. The DNA binding sites of “MYB DNA-bind 6” in Group 2 contained 11 residues and there were one and ten residues in α-helix1 and α-helix3, respectively (Fig. 4). The MYB transcription factors containing “MYB DNA-bind 6” in Group 1 were distributed in Clade I of the phylogenetic tree (Fig. 2), and contained the binding site “F-WS/T-R-PTD-RDR-RN”. The MYB transcription factors containing “MYB DNA-bind 6” in Group 2 were distributed in Clades II, III, IV, X, and XI (Fig. 2). The “D” in α-helix1 and “W” in α-helix2 were absolutely conserved. In addition, the C- and N-termini of the “MYB DNA-bind 6” domain contained the same conserved amino acid motif “W^T^/_S_-EEDE-L”, which was also present in the N-termini of the amino acid sequences of “MYB DNA-bind”.

### Promoter analysis of 79 *MYB* genes from six sequenced *Trichoderma* genomes

**Figure 5:**
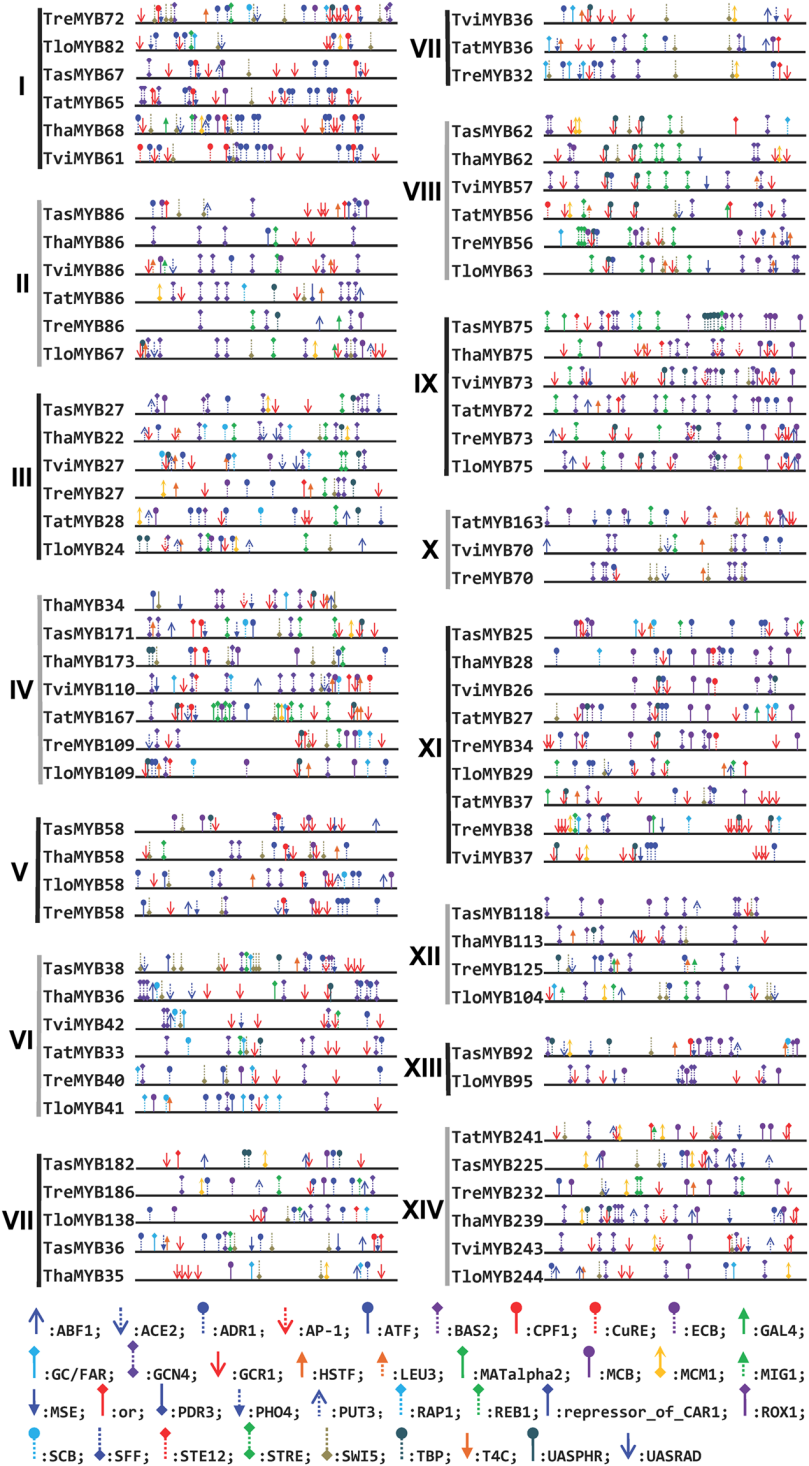
Promoter analysis of 79 *MYB* genes*. T. asperellum*, *T. harzianum*, *T. virens*, *T. reesei*, *T. atroviride*, and *T. longibrachiatum* are abbreviated as Tas, Tha, Tvi, Tat, Tre and Tlo. The name of each MYB transcription factor is made up of three parts, including the abbreviation of the species name, MYB, and the molecular weight of the protein. The different colored markers indicate predicted different cis-motifs.

The 1,000 bp upstream sequences of the 79 *MYB* genes from the six sequenced *Trichoderma* genomes were cloned. Using SCPD analysis, motifs were predicted and their locations were marked in the promoter regions of the *MYB* genes (Fig. 5). Most of the motifs were closely related to stress responses and detoxification, including GCR1, GCN4, ADR1, STRE, HSTF, PHO4, GC/FAR, PDR3, ABF1, CPF1, and so on. In particular, there were 240 GCR1s and 214 GCN4s motifs in the promoter regions of the 79 *MYB* genes. In addition, motifs GCR1, ADR1, HSTF and ABF1 are closely related to G-proteins and their receptors which are involved in sensing the oligopeptides secreted by phytopathogens^30^, so these motifs play an important roles in upregulation of disease-resistant genes, synthesis of secondary metabolites and hyperparasitism. In addition, the type, density, and location of the motifs in MYB promoter regions were closely related to the position of the corresponding MYB transcription factors in the phylogenetic tree. The promoter sequences of 6 *MYBs* in Clade I contained many GCR1s and ADR1s motifs (average 4.8 and 6.2, respectively) (Fig. 5), which can improve the binding efficiency of transcription factors with both motifs, so these MYBs were closely related to signal transduction of *Trichoderma* in biocontrol process. The promoter sequences of 12 *MYBs* in Clade II and VIII contained more GCN4s (average 4.5) and STRE (average of 2.80) motifs than the other *MYBs* respectively, so they could mainly responded to biotic and abiotic stresses, and further upregulated defense response genes in *Trichoderma*. These STRE motifs were distributed mainly from −600 to −900 bp. All above indicated MYB transcription factors played important roles in *Trichoderma* adapting environment and survival.

### The construction of *Populus* transformants and *TasMYB36* transcription detection

**Figure 6:**
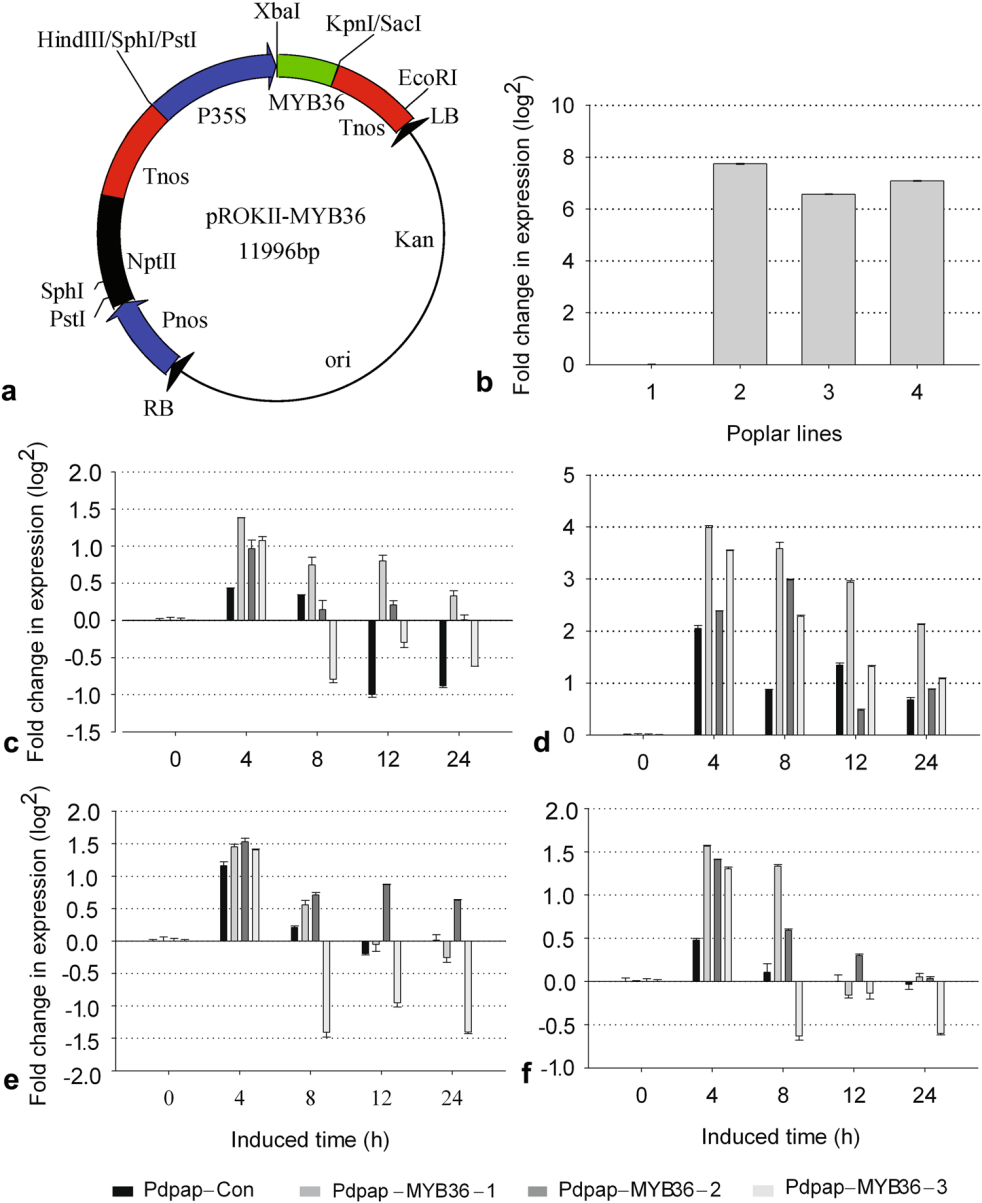
The construction of expression vector pROKII-TasMYB36, and transcription detection of *TasMYB36* gene and plant hormone signal genes in PdPap-TasMYB36s. (**a**) The recombinant vector pROKII-MYB36. (**b**) Transcription detection of *TasMYB36* gene in PdPap-TasMYB36s, 1–4: PdPap-Con, PdPap-TasMYB36-1, PdPap-TasMYB36-2, and PdPap-TasMYB36-3. (**c**,**d**) The transcription analysis of *NPR1* and *PR1* in the sialic acid (SA) pathway. (**e**,**f**) The transcription analysis of *JAR1* and *MYC2* in the jasmonic acid (JA) pathway.

The plant transgenic vector pROKII-MYB36 was constructed (Fig. 6a) and transgenic *Agrobacterium tumefaciens* EHA105-MYB36 were obtained. The leaves of PdPap poplar were then infected with EHA105-MYB36 and 15 transformants were obtained by selecting with Kanamycin (50 mg/L); however, uninfected leaves did not generate resistant buds. PCR detection indicated that *TasMYB36* from *T. asperellum* was integrated successfully in the *Populus* genome and three transformants (PdPap-TasMYB36s) were chosen for subsequent experiments. The qRT-PCR analysis showed that the *TasMYB36* was transcribed in three PdPap-TasMYB36s, but not in PdPap-Con (Fig. 6b).

### Transcription analysis of PdPap-TasMYB36s hormone signal transduction genes related to biotic stress responses

The SA and JA pathways (KEGG: map04075) are two important signal transduction pathways related to stress responses. In the SA pathway, the *PR1* as pathogenesis related gene can respond to biotic stresses and improve the antifungal ability of organisms, and the NPR1 protein could be the sole sensor from SA to *PR* gene expression in SA hormone signal transduction pathway^31^. In our study, the transcription of *NPR1* and *PR1* were upregulated in all three PdPap-TasMYB36s at 4 h under *A. alternata* stress, especially in PdPap-TasMYB36-1, in which the transcription of *NPR1* and *PR1* were upregulated by 2.60 (2^1.38^)-fold and 15.89 (2^3.99^)-fold respectively, and the transcription level of *NPR1* and *PR1* showed a positive correlation with each other (Fig. 6a,b). In the JA pathway, the JAR1 as jasmonic acid-amino synthetase could synthesize jasmonic acid (JA). JASMONATE ZIM-DOMAIN (JAZ) proteins could repress the expression of JA-response gene MYC2. However the perception of bioactive JA by the F-box protein COI1 (coronatine-insensitive protein 1) triggers the SCF^COI1^/ubiquitin-dependent degradation of JAZ, and further enhance the expression of transcription factor gene *MYC2*. MYC2 can up-regulate others genes involved in a wide range of biological processes, including plant defense, secondary metabolism, and growth control^32,33^. In our study, the *JAR1* transcription in the PdPap-TasMYB36s was upregulated compared with PdPap-Con (Fig. 6c). Especially, *JAR1* in PdPap-TasMYB36-2 was upregulated to the highest level, by 2.89 (2^1.53^)-fold. The transcription of *MYC2* in the PdPap-TasMYB36s was also upregulated compared with that in PdPap-Con. Especially, *MYC2* in PdPap-TasMYB36-1 was upregulated to the highest level, by 2.95 (2^1.56^)-fold (Fig. 6d).

### Antifungal ability of the PdPap-TasMYB36s under *A. alternate* stress

**Figure 7:**
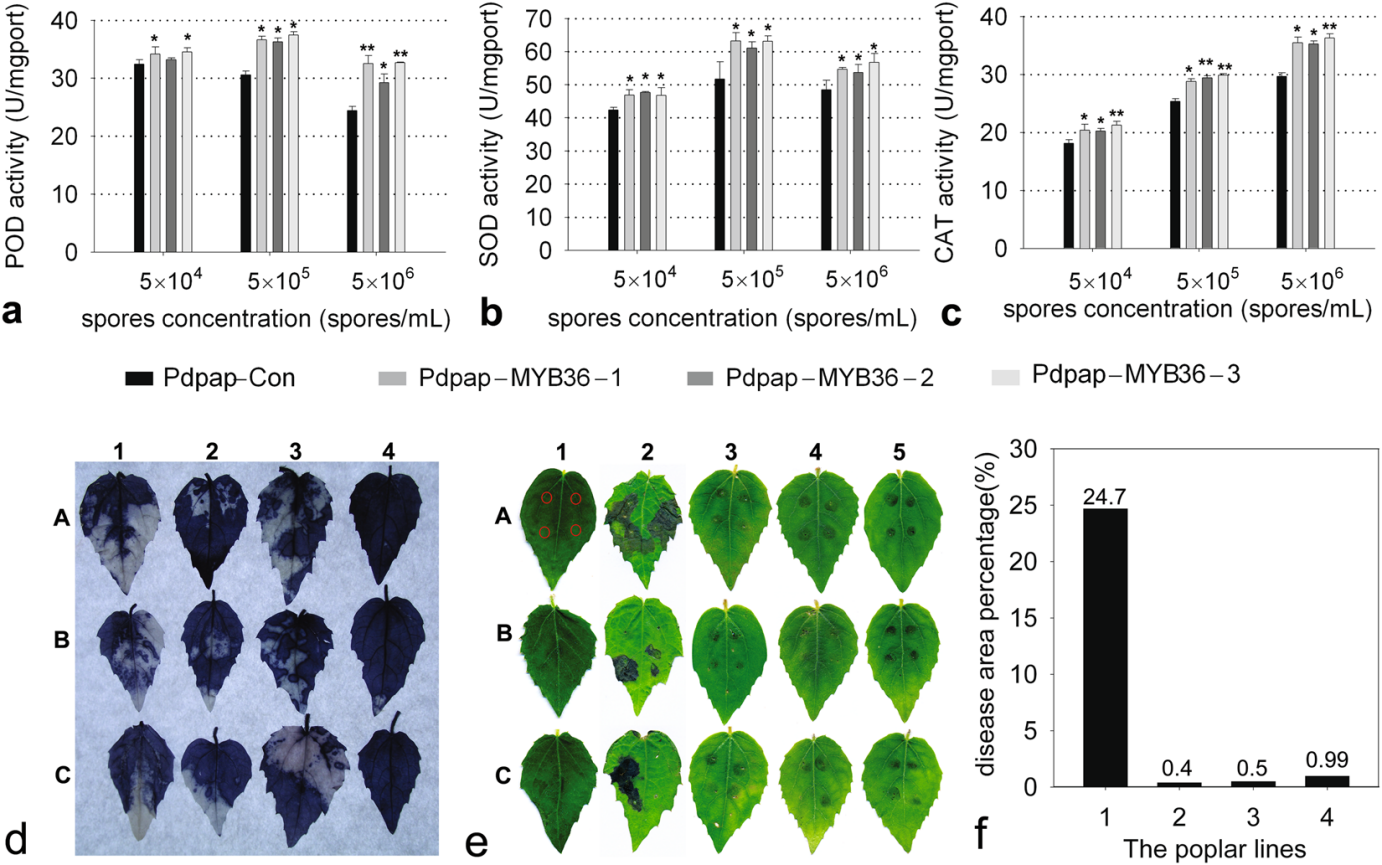
The ability of transformants PdPap-TasMYB36s to resist infection by the pathogenic fungus *A. alternate*. (**a**–**c**) The POD, SOD, and CAT activities under *A. alternate* stress, respectively (student *t*-test p < 0.05). (**d**) The reactive oxygen content under *A. alternate* stress, 1–4: PdPap-Con, PdPap-TasMYB36-1, PdPap-TasMYB36-2, and PdPap-TasMYB36-3, respectively, A-C: three repeated experiments. (**e**) The ability of transformant PdPap-TasMYB36s to resist *A*. *alternate* infection. A1-C1 and A2-C2: leaves of the PdPap-Con. A3-C3, A4-C4, and A5-C5: the leaves of PdPap-TasMYB36-1, PdPap-TasMYB36-2, and PdPap-TasMYB36-3, respectively. The leaves A1-C1 were not inoculated with *A*. *alternate*, the others were inoculated with 5 × 10^6^ spores/mL *A*. *alternate* for 4 d. The inoculated positions on the leaves are shown on leaf A1. The three repeats of each poplar line were designated as A, B, and C. (**f**) The percentage of disease spot area, 1–4: PdPap-Con, PdPap-TasMYB36-1, PdPap-TasMYB36-2, and PdPap-TasMYB36-3.

Following the interaction of the PdPap-TasMYB36s with *A. alternate*, the antifungal ability of PdPap-TasMYB36s was studied by assessing the change in the POD, SOD, and CAT activities. SOD enzymes are responsible for decomposing the superoxide anion radical (O_2_−) to H_2_O_2_ and O_2_. peroxidase (POD) and catalase (CAT) further decompose H_2_O_2_^34^. The POD activities of the three PdPap-TasMYB36s were higher than those of PdPap-Con and reached the highest level, approximately 37.0 U, at 5 × 10^5^ spores/mL. In addition, the POD activities of PdPap-Con decreased consistently, but the fluctuation of POD activities of PdPap-TasMYB36s was not obvious as the concentration of *A. alternate* spores increased (Fig. 7a).The POD activities of the three PdPap-TasMYB36s were higher than those of PdPap-Con and reached the highest level, approximately 37.0 U, at 5 × 10^5^ spores/mL. In addition, the POD activities of PdPap-Con decreased consistently, but the fluctuation of POD activity was not obvious as the concentration of *A. alternate* spores increased (Fig. 7a). The SOD activity of all PdPap-TasMYB36s was higher than that of PdPap-Con at all spore concentrations, and its activity reached the highest at 5 × 10^5^ spore/mL in all PdPap-TasMYB36s. At this spore concentration, the SOD activity of PdPap-Con was 51.7 U and that of PdPap-TasMYB36s were at least 61.1 U (Fig. 7b). The CAT activity of PdPap-Con ranged from 18.1 to 29.7 U, and the CAT activities of PdPap-TasMYB36s were higher at all spore concentrations, ranging from 20.2 to 36.3 U. The CAT activities increased with the increasing spore concentration (Fig. 7c).

The antifungal ability of PdPap-TasMYB36s was also studied using the ROS contents of poplar leaves under *A. alternate* stress (Fig. 7d). NBT staining revealed that the ROS content was lower in the PdPap-TasMYB36s leaves (smaller extent of blue staining) compared with that in the PdPap-Con leaves (larger extent of blue staining). PdPap-TasMYB36-2 had more blue staining compared with the other PdPap-TasMYB36s.

After PdPap-TasMYB36s and PdPap-Con were infected with *A. alternate*, the disease spots on leaves of PdPap-TasMYB36s were smaller (diameter: approximately 1–2 mm), showed regular circles, and the average of their area percentage was 0.63%, while the disease spots on the PdPap-Con leaves were larger (average diameter: 5–7 mm) and spread around, forming irregular shapes, even connected together (Fig. 7e), and the average of their area percentage was 24.7%. The leaf mortalities in the PdPap-TasMYB36-1, PdPap-TasMYB36-2, and PdPap-TasMYB36-3 were 33.3, 50, and 41.7%, respectively, whereas that in PdPap-Con reached 83.3%.

## Discussion

MYB transcription factors are involved in a plant’s response to many diseases^24,35^, for example, stripe rust fungus, ear heading and *Bipolaris sorokiniana* in wheat^36^; and *Phakopsora pachyrhizi* in soybean^37^. However, few *MYB* genes of the biocontrol species *Trichoderma* have been studied.

In the present study, the functions of the *TasMYB36* gene from *T. asperellum* CBS433.97 were explored. The treatment of *T. asperellum* with four out of five phytopathogen fermentation broths caused the transcription of *TasMYB36* to increase (Fig. 1b–e), especially in response to *A. alternate*; however, it was downregulated in response to *S. sclerotiorum* stress (Fig. 1f). Moreover, there were ten kinds of motifs related to stress response in *TasMYB36* gene (Fig. 5), especially motifs ADR1 (n = 4), HSTF (n = 1), GCR1 (n = 1), and ABF1 (n = 1) are closely related to G-proteins and their receptors which are involved in sensing the oligopeptides secreted by phytopathogens. Combined results of the differential expression (Fig. 1) and promoter analysis (Fig. 5), it could be putative reason that *T. asperellum* sensed pathogenic toxin, then signal molecular promoted upstream transcription factors bind to the motifs in the promotor of *TasMYB36*, further induced the expression of *Trichoderma* biocontrol genes, which improved the ability of *T. asperellum* to resist phytopathogen. So it was considered that TasMYB36 played important roles in biocontrol process of *T. asperellum*. Furthermore, it was reported that an R2R3 MYB transcription factor in wheat mediates host resistance to *Bipolaris sorokiniana* ^38^ and a sorghum MYB transcription factor enhances resistance against leaf blight in maize^24^. Our results further indicated that *TasMYB36* could respond to different pathogens. *TasMYB36* showed different transcription levels under the five biotic stresses (Fig. 1), possibly because different types and levels of toxins were generated by the different pathogens, or that the resistance was associated with the synergistic action of many genes, and a single gene only responded to some stresses in different degrees.

To investigate the properties of homologous genes of *TasMYB36*, 79 *MYB* genes were obtained from six sequenced *Trichoderma* genomes. Most MYB transcription factors belonged to 1R and 2R MYB; only ThaMYB34 was a 3R-MYB (Table 1). In plants, only a few MYB transcription factors are 3R and 4R MYB proteins, most of them are 1R and 2R MYBs^20,28^, indicating the MYB transcription factors in fungal and higher plant genomes could have similar structures, and that 1R and 2R MYB might be the most widespread form of MYB transcription factors in organisms. Furthermore, the phylogenetic analysis (Fig. 2) divided the MYB transcription factors into 14 clades and their distribution was closely related to their type, molecular weight, pI, and number of introns (Table 1), which indicated the MYB proteins in same clades were conserved and might have similar functions, whereas those in different clades might have different in functions or have roles in different pathways. Plant MYB transcription factors from different phylogenetic clades also had different functions, for example in the immune response, hormone signal transduction, and secondary metabolite biosynthesis^39^. So it’s speculated that the MYB transcription factors in *Trichoderma* play important roles in many physiological processes. The type 8 (B_6_B_4_) and type 3 (B_6_) MYB proteins were distributed in Clade IV and contained a similar “MYB DNA-bind 6” domain, suggesting the both type MYB proteins might have shared the same ancestor.

The alignment analysis showed the amino acid sequences of “MYB DNA-bind” (Fig. 3) and “MYB DNA-bind 6” (Fig. 4) in the same phylogenetic clade were relatively conserved, and those from different clades had obvious differences, which would explain why the MYB proteins in the same clade are likely to share similar properties (Table 1). The DNA binding sites of MYB transcription factors in Clade I and XII had obvious differences to those in other MYB transcription factors; therefore, we speculated that the MYB transcription factors in Clade I and XII might recognize different cis-motifs and regulate different resistance genes, compared with those in the other clades. In plants, the MYB DNA binding domains contain three regularly spaced tryptophan (W) residues that together form a hydrophobic core^40^; however in *Trichoderma*, the third “W” residues generated mutant (corresponding location marked with asterisk) and are replaced by hydrophilic tyrosine residues (Y). Thus the three-dimensional structures of MYB transcription factors in plants and fungi could be different, which requires further study. Most residues of MYB DNA binding sites (Figs 3 and 4) were distributed in α-helix3, suggesting that it is important to the function of MYB transcription factors. Previous studies showed that the third α-helix of the DNA binding domain was major DNA-recognition α-helix and bound to the major DNA groove. In MYB transcription factors with two or more MYB DNA binding domains, DNA binding involves synergistic DNA recognition by the third α-helix of the individual MYB repeats^15,41^. The “E” in α-helix1 of “MYB DNA-bind” (Fig. 3b) and the “D” in α-helix1 of “MYB DNA-bind 6”(Fig. 4b) are relatively conserved in *Trichoderma*, and plants also have the conserved “E” or “D” residues; therefore, we hypothesized that both residues might play important roles in the function and structure of MYB transcription factors of fungi. This too will require further study to confirm the hypothesis. In addition, the “MYB DNA-bind” (Fig. 3b) and “MYB DNA-bind 6” (Fig. 4b) domains contained the same conserved sequence “W^T^/_S_-EEDE-L”. The MYB DNA binding domains of 30 types of plants also contained the conserved sequence “W^T^/_S_-EEDE-L”^28,29^, indicating that the DNA binding domains of plants and *Trichoderma* might have similar functions and are derived from the same ancestor.

The promoter analysis (Fig. 5) showed the motifs in the promoter regions of MYB transcription factors in the same clade share similar type, density, and location. This further indicated that MYB transcription factors in same clade would share similar functions and be regulated by the same upstream genes; however, those in different clades might function in different pathways. In addition, the identified motifs are closely related to stress responses and detoxification, which suggested that MYB transcription factors could improve stress resistance of *Trichoderma*, allowing it to survive in harsh environments. In particular, motifs GCR1, ADR1, HSTF, and ABF1 are closely related to G-proteins and their receptors. G-protein receptors act as sensors for oligopeptides, and are involved in sensing the oligopeptides secreted by phytopathogens^30^, which further stimulates the synthesis and secretion of secondary metabolites, and the expression of disease-resistance genes in *Trichoderma*. The promoter analysis showed that MYB transcription factors play an important role in the defense response of *Trichoderma*.

Furthermore, after the *TasMYB36* gene was integrated into the genome of PdPap poplar, four genes involved in SA and JA hormone signal transduction pathways were upregulated under *A. alternate* stress (Fig. 5). A previous study proved that plants produce and perceive JA signals, which induce the degradation of JASMONATE-ZIM-Domain (JAZ) proteins and derepress the JAZ-repressed transcription factors to regulate diverse aspects of the defense response^42^. *TaPIMP1* (an *R2R3 MYB*) in wheat upregulates a subset of defense- and stress-related genes in the SA signaling transduction pathway, for example *PR1a* ^38^. Combined with those of previous studies, our results indicated that *TasMYB36* could enhanced the SA and JA hormone signal transduction pathways, further upregulating the defense and stress-related genes, and improved the resistance of poplar to biotic stress. In addition, POD, SOD, and CAT, as ROS-scavenging enzymes, could decrease ROS levels of plant and improve their fungal resistance^43^. In addition, it was reported that wheat R2R3-MYB transcription factors increased the resistance of tobacco to pathogens and abiotic stress, which may participate in the ROS scavenging pathway and elevated levels of ROS-scavenging enzymes^25^. In our study, the activities of POD, SOD, and CAT were higher in the transformants than that in control under *A. alternate* stress (Fig. 7c). NBT staining showed that the ROS content was lower in the leaves of the transformants compared with that in control leaves (Fig. 7d). In a previous study, following the overexpression of LeAN2 (an *R2R3-MYB* gene) in tomato, NBT staining showed that the ROS level was lower in transgenic tomatoes than in the control under heat, chilling, and oxidative stresses^44^. Combining our results with those of previous studies, the *TasMYB36* gene from *T. asperellum* could also enhance the activities of POD, SOD, and CAT and decrease the accumulation of ROS, further improving the resistance of PdPap-TasMYB36 transformants to biotic stresses. After infection with *A. alternate* spores, the leaves of the PdPap-TasMYB36s transformants developed smaller disease spots, the average percentage of the disease spot area was 0.63% (PdPap-Con 24.7%), and showed lower morbidity (average 41.7%) compared with PdPap-Con (83.3%) (Fig. 7e). In previous studies, under *Ralstonia solanacearum* stress, the brown necrotic areas in the injection zone of wild-type tobacco expanded to cover over half of the inoculated leaf. The disease index was as high as 97.6%, whereas the symptoms on transgenic tobacco transformed heterologously with a wheat MYB gene were much less severe: their disease index ranged from 8.3 to 18.7%^25^. These previous reports supported our observations that the *TasMYB36* gene of *T. asperellum* could enhance the resistance of PdPap-TasMYB36 transformants to *A. alternate* stress.

In conclusion, *TasMYB36* is an excellent disease-resistance gene that could endow *Trichoderma* with the ability to resist biotic stresses. The phylogenetic and alignment analysis showed the conservation of MYB transcription factors and confirmed the presence of many motifs related to stress response in their promoter regions. Following heterologous transformation into PdPap poplar, the *TasMYB36* gene enhanced hormone signal transduction pathways and upregulated the expression of disease-resistance and defense-response genes, further stimulating the resistance of the transformants to biotic stresses, for example *A. alternate*. Furthermore, we hypothesized that the other MYB transcription factors in *Trichoderma* might play important roles in resisting environmental stresses and should be the subjects of further study.

## Materials and Methods

### Strains, plasmids, and plant materials

*T. asperellum* CBS433.97 was obtained from the Agricultural Culture Collection of China and the phytopathogenic fungi *Alternaria alternate* was used for the pathogen-poplar interaction assay. *Agrobacterium tumefaciens* EHA105 and vector pROKII (Vaughan *et al*. 1987) were used for the genetic transformation of *Populus davidiana* × *P. alba* var. *pyramidalis* Louche (PdPap poplar). PdPap poplar seedlings were cultured aseptically in liquid rooting medium (MS medium with 1-Naphthaleneacetic acid (NAA) 0.25 mg/L and Sucrose 20 g/L) or differential medium (MS medium with NAA 0.05 mg/L, 6-Benzylaminopurine (6-BA) 0.5 mg/L, Sucrose 20 g/L and Agar 8 g/L) at 25 °C.

### The cloning of the *TasMYB36* gene and its responses to five kinds of fermentation broths stresses

The *TasMYB36* was cloned using the sense primer (5′-ATGACACCTCACGTTCCAGA-3′) and the antisense primer (5′-TTATGCCAGTCTTCGCCTATG-3′). The transcription of *TasMYB36* from *T. asperellum* CBS433.97 was detected by qRT-PCR under five biotic stresses: fermentation broths from *A. alternate*, *C. chrysosperma*, *F. oxysporum*, *R. solani* and *S. sclerotiorum*, respectively. The spores of *T. asperellum* were inoculated into 200 mL of 1/4 PD (Potato Dextrose) at a final concentration of 1 × 10^4^ spores/mL and cultured at 28 °C with continuous shaking at 200 rpm for 48 h. The mycelia were filtered, washed, and cultured in minimal medium (MM) for 2 h, and then transferred into the different MM inducing media (including the five fermentation broths mentioned above) for 48 h. For each inducing condition, mycelia were collected at 0, 4, 8, 12, 24, and 48 h post-induction. At each time point, 25 mL mycelium cultures per flask were collected, the biomass obtained in the three replicates and three 25 mL mycelium from three flasks under the same conditions were mixed and then stored at −80 °C. Total RNA was extracted from mycelia using the Trizol reagent (Invitrogen, USA), digested with DNaseI (Promega, USA), and reverse transcribed into cDNA using a PrimeScript RT Kit (Takara, Japan), according to the manufacturer’s instructions. The transcription level of *TasMYB36* in *T. asperellum* was detected by qRT-PCR and calculated according to the 2^−ΔΔCt^ method^45,46^, using cDNA as the template and *Actin*(T) as the reference gene. Three qRT-PCR replicates were performed for each cDNA sample. Primers for qRT-PCR (Supplemental Table 1) were designed using Primer 6.0 software (PREMIER Biosoft, USA).

### Analysis of 79 MYB transcription factors from six sequenced *Trichoderma* genomes

Seventy-nine *MYB* genes were identified in the genomes of six sequenced *Trichoderma*, including *T. asperellum* CBS433.97, *T. harzianum* CBS226.95, *T. virens* Gv29-8, *T. atroviride*, *T. reesei*, and *T. longibrachiatum* ATCC18648 (http://genome.jgi.doe.gov/pages/tree-of-life.jsf). The conserved domains of the 79 MYB proteins were predicted using the BlastP program and analyzed using the Pfam program (http://pfam.sanger.ac.uk/). Furthermore, 1,000 bp sequences upstream of the transcription start site of these *MYB* genes were obtained from *Trichoderma* genomes and the motifs in the presumed promoters were predicted using the SCPD promoter analysis website (http://rulai.cshl.edu/SCPD/).

### Phylogenetic and alignment analysis of MYB transcription factors from six sequenced *Trichoderma* genomes

A phylogenetic tree was constructed with amino acid sequences of 79 MYB transcription factors using the Neighbor-Joining method in MEGA6.0. And the genetic distances of 79 MYB was calculated with MEGA6.0. The tree is drawn to scale, with branch lengths in the same units as those of the evolutionary distances used to infer the phylogenetic tree. The evolutionary distances were computed using the Poisson correction method and were in the units of the number of amino acid substitutions per site. All positions containing gaps and missing data were eliminated. There were 72 “MYB DNA-binding” and 30 “MYB DNA-binding 6” domains found in the 79 MYB proteins, and alignment analysis of both types of DNA-binding domains was carried out using Clustal Omega (http://www.ebi.ac.uk/services/proteins) and the sequence logo was drawn using Weblogo (http://weblogo.berkeley.edu/logo.cgi).

### Construction of the plant expression vector pROKII-MYB36 and *Populus* transformation

To further detect the functions of *TasMYB36*, the gene was cloned by PCR using the sense primer (5′-ATCGTCTAGAATGACACCTCACGTTCCAGA-3′) containing an *Xba*I site and the antisense primer (5′-CGATGGTACCTTATGCCAGTCTTCGCCTATG-3′) containing a *Kpn*I site. The PCR product of *TasMYB36* and pROKII vector were double-digested with *Xba*I and *Kpn*I, respectively, and ligated to generate vector pROKII-MYB36 (Fig. 6a). *TasMYB36* was transformed into PdPap poplar using the *Agrobacterium tumefaciens*-mediated transformation system. Explants of PdPap poplar were cultured in differential medium containing 50 mg/L Kanamycin (used to select resistant buds), and 300 mg/L Cefotaxime sodium and 300 mg/L Ampicillin (used to eliminate *Agrobacterium tumefaciens*) at 25 °C for 15 d. Kanamycin-resistant buds were cut down and cultured in differential medium containing 30 mg/L Kanamycin for propagation. When the propagated seedlings grew to 3 cm in height, they were rooted in liquid rooting medium containing 30 mg/L Kanamycin, and finally transplanted into 10 cm pots containing a mixture of peat soil and vermiculite (5:1).

### Transcription analysis of hormone signal transduction genes of PdPap-TasMYB36s

The transcription of hormone signal transduction genes (*NPR1*, *PR1*, *JAR1*, and *MYC2*) in the polar transformants (PdPap-TasMYB36s) under *A. alternate* stress were detected by qRT-PCR. *A. alternate* was inoculated in PD (Potato Dextrose) medium at 28 °C with continuous shaking at 200 rpm for 8 d and the fermentation broth was collected. The control PdPap-Con and three PdPap-TasMYB36s were induced in the fermentation broth for 24 h, respectively. Thereafter, 1 g of poplar leaves was collected from PdPap-Con and the three PdPap-TasMYB36s at 6, 12, and 24 h, and stored at −80 °C. Total RNA was extracted from poplar leaves using the cetyl trimethylammonium bromide (CTAB) method and reverse-transcribed into cDNA. The primers for qRT-PCR were the same as those used in our previous study^47^. The qRT-PCR method was in accordance with description in the section of “The responses of the *TasMYB36* gene under five biotic stresses”.

### The detection of the ROS content and activity analysis of POD, SOD, and CAT

The PdPap-TasMYB36s and PdPap-Con were transplanted into soil and cultured for 40 d. Then, three concentrations of *A. alternate* spores suspensions were set, including 5 × 10^6^, 5 × 10^5^, and 5 × 10^4^ spores/mL, and each individual plant was treated with 50 mL of *A. alternate* spores suspension in the three replicates. After *A. alternate* spores and poplar were co-cultured for 5 d in soil, the top fifth leaves of PdPap-TasMYB36 and PdPap-Con were obtained and weighed using an analytical balance, before being ground in a mortar on the ice and centrifuged at 1100g for 10 min, respectively. Following the POD, SOD and CAT in supernatants were reacted for 30 min at 37 °C with the oxide respectively, the residual oxide in supernatants were reduced by another reductant and the supernatants showed a color by the interaction between reduzate and chromogenic agent. Finally, the content of reduzate was detected using spectrophotometer, and further worked out the activities of POD, SOD and CAT with the kit manufactured by nanjing jiancheng bioengineering institute (POD: A084-3, SOD: A001-1, and CAT: A007-1). The ROS levels in the leaves of PdPap-TasMYB36s were also analyzed. PdPap-TasMYB36s and PdPap-Con were cultured for 13 d in liquid rooting medium and the phytopathogen *A. alternate* was cultured in Potato Dextrose (PD) medium for 8 d. *A. alternate* mycelia were allowed to interact with the roots of the PdPap-Con and PdPap-TasMYB36s in sterile water for 48 h at 25 °C. Subsequently, the leaves of the PdPap-Con and PdPap-TasMYB36s were stained with 1 mg/mL nitroblue tetrazolium (NBT) overnight and destained in a solution containing 75% ethanol and 5% glycerol in a boiling water bath for 5 min.

### The antifungal ability of PdPap-TasMYB36s against *A. alternate*

*A. alternate* was cultured in PDA medium at 28 °C, the spores were collected after 20 d and diluted to 5 × 106 spores/mL with sterile water. Leaves from PdPap-TasMYB36s and PdPap-Con were then infected by injection (two sites on either side of the main vein) with 5 μL of spore suspension, and cultured at 25 °C on wet filter paper in a petri dish. The relative area of disease spots were calculated by Chalkiness 1.0 program. Firstly, the infected leaves were scanned for digital images. Following the images inputted into Chalkiness 1.0 program, the location and profile of disease spots were recognized accurately, then the relative area of disease spots was evaluated^48^.

## Electronic supplementary material

Supplemental Table 1

Supplemental Table 2

Supplemental Table 3

Supplemental Table 4

Supplemental Table 5

Supplemental Table 6

Supplemental Table 7
