# Supplementary material for: Properties analysis of transcription factor gene TasMYB36 from Trichoderma asperellum CBS433.97 and its heterogeneous transfomation to improve antifungal ability of Populus
Source: Sci Rep. 2017 Oct 9;7:12801. doi: 10.1038/s41598-017-13120-w (PMC5634415; doi:10.1038/s41598-017-13120-w)
Supplement: Supplementary file 1 — Supplemental Table 1 [file 41598_2017_13120_MOESM1_ESM.pdf]

Properties analysis of transcription factor gene *TasMYB36* from *Trichoderma asperellum* CBS433.97 and its heterogeneous transfomation to improve antifungal ability of *Populus*

Shida Ji1, 2, Zhiying Wang1, Jinjie Wang1, Haijuan Fan1, Yucheng Wang1, Zhihua Liu1*

Supplemental Table 1 The primers for qRT-PCR

Genes	Primers	Sequences (5'–3')	Tm/°C
***TasMYB36*(T)**	MYB3L	TCACCCTCCCTCTCACAACACA	58.9
	MYB3R	ATGCCAGTCTTCGCCTATGAGC	58.9
***Actin*(T)**	ActinL	AGGCAACCTTCTCGCCAACG	59.0
	ActinR	TCGCTTCTCGACAATGCCAACT	58.9
***Actin*(P)**	AP-5	TTCCGTTGCCCTGAGGTCCTAT	59.1
	AP-3	TCAGGAGGAGCAACCACCTTGA	59.3

T: Genes from *T. asperrellum*. P: Gene from Pdpap poplar
