# Supplementary material for: Properties analysis of transcription factor gene TasMYB36 from Trichoderma asperellum CBS433.97 and its heterogeneous transfomation to improve antifungal ability of Populus
Source: Sci Rep. 2017 Oct 9;7:12801. doi: 10.1038/s41598-017-13120-w (PMC5634415; doi:10.1038/s41598-017-13120-w)
Supplement: Supplementary file 6 — Supplemental Table 6 [file 41598_2017_13120_MOESM6_ESM.pdf]

Properties analysis of transcription factor gene *TasMYB36* from *Trichoderma asperellum* CBS433.97 and its heterogeneous transfomation to improve antifungal ability of *Populus*

Shida Ji1, 2, Zhiying Wang1, Jinjie Wang1, Haijuan Fan1, Yucheng Wang1, Zhihua Liu1*

Supplemental Table 6 The genetic distances between 12 MYBs amino acid sequences from *Trichoderma atroviride* genome

	1	2	3	4	5	6	7	8	9	10	11	12
1		0.297	0.235	0.225	0.269	0.187	0.200	0.268	0.318	0.291	0.232	0.251
2	2.890		0.192	0.223	0.189	0.238	0.216	0.186	0.174	0.216	0.234	0.245
3	2.516	2.110		0.258	0.200	0.223	0.132	0.186	0.262	0.227	0.210	0.260
4	2.344	2.398	2.580		0.254	0.275	0.239	0.225	0.218	0.231	0.235	0.287
5	2.723	2.069	2.153	2.649		0.237	0.185	0.194	0.162	0.264	0.228	0.253
6	2.069	2.580	2.293	2.723	2.455		0.193	0.199	0.247	0.270	0.200	0.293
7	2.153	2.293	1.527	2.455	2.030	2.153		0.125	0.189	0.238	0.249	0.255
8	2.649	2.030	2.030	2.398	2.110	2.153	1.417		0.216	0.188	0.160	0.272
9	2.986	1.956	2.580	2.344	1.887	2.516	2.153	2.398		0.211	0.218	0.251
10	2.890	2.344	2.344	2.344	2.723	2.723	2.455	2.030	2.244		0.260	0.256
11	2.398	2.455	2.244	2.398	2.455	2.110	2.516	1.792	2.293	2.580		0.323
12	2.580	2.580	2.580	2.723	2.516	2.803	2.516	2.649	2.580	2.649	3.091	

The number of amino acid substitutions per site between sequences are shown below the diagonal. Standard error estimates are shown above the diagonal and were obtained by a bootstrap procedure (1000 replicates). The analysis involved 12 MYBs amino acid sequences. All positions containing gaps and missing data were eliminated. There were a total of 198 positions in the final dataset. Evolutionary analyses were conducted in MEGA6 program. 1-12: TatMYB28T6, TatMYB86T6, TatMYB33T5, TatMYB65T7, TatMYB163T3, TatMYB37T3, TatMYB36T1, TatMYB241T5, TatMYB56T4, TatMYB167T3, TatMYB27T1, and TatMYB72T1.
